# Dietary Intake of Coumarins and Furocoumarins through Citrus Beverages: A Detailed Estimation by a HPLC-MS/MS Method Combined with the Linear Retention Index System

**DOI:** 10.3390/foods10071533

**Published:** 2021-07-02

**Authors:** Adriana Arigò, Francesca Rigano, Marina Russo, Emanuela Trovato, Paola Dugo, Luigi Mondello

**Affiliations:** 1 Department of Chemical, Biological, Pharmaceutical and Environmental Sciences, University of Messina, 98168 Messina, Italy; aarigo@unime.it (A.A.); marina.russo@unime.it (M.R.); pdugo@unime.it (P.D.); lmondello@unime.it (L.M.); 2 Chromaleont s.r.l., c/o Department of Chemical, Biological, Pharmaceutical and Environmental Sciences, University of Messina, 98198 Messina, Italy; emanuela.trovato@chromaleont.it; 3 Unit of Food Science and Nutrition, Department of Medicine, University Campus Bio-Medico of Rome, 00128 Rome, Italy; 4 BeSep s.r.l., c/o Department of Chemical, Biological, Pharmaceutical and Environmental Sciences, University of Messina, 98168 Messina, Italy

**Keywords:** beverages, *Citrus*, coumarins, furocoumarins, polymethoxyflavones, linear retention indices, liquid chromatography, tandem mass spectrometry, multiple reaction monitoring, quality control

## Abstract

Official regulations concerning the maximum number of substances in food are introduced as a consequence of possible adverse effects, after oral administration. In this regard, analytical methods are necessary in order to determine specific targets. Among oxygen heterocyclic compounds (OHCs, that are furocoumarins, coumarins and polymethoxyflavones), only coumarin is subject to restriction by the *Regulation (EC) No 1334/2008* of the European Parliament. Furocoumarins are known for their phototoxicity and other side effects due to their dietary intake; however, an official limit about the maximum content of these compounds in food is still missing. The lack of information about the real amount of these compounds in food is responsible for the conflicting opinions about the introduction of an official limit. The HPLC-MS/MS method here proposed, in combination with the linear retention index system, represents an innovative analytical strategy for the characterization of OHCs in citrus beverages. Several types of drinks were analysed in order to quantify 35 OHCs in total. This method is suitable for the quality control of OHCs in food and the obtained results may be considered as informative data useful for the regulatory authorities in the emission of new opinions and for a potential new regulation in this field.

## 1. Introduction

Furocoumarins (FCs) are secondary plant metabolites produced in response to attack by pests and to stressful challenges. They are particularly prevalent in the Apiaceae, Umbelliferae [1,2], Fabaceae and Rutaceae [3] families. Edible fruits and vegetables may also contain FCs, e.g., parsnips (Pastinaca sativa), in which the highest content of FCs was detected [4].

Among Rutaceae botanical species, FCs are characteristic compounds of citrus peel [5]. Recently, 61 citrus species, chosen as representatives of the genetic diversity of the species, were analysed to determine the contents of these compounds in peels and pulps [6]. Among them, a large amount of FCs was found in grapefruit (*Citrus Paradisi*), where they are responsible for the so called “grapefruit juice effect”, which consists of the dangerous interaction with drug metabolism, specifically in the inhibition of the enzymes involved in the catabolism of some medications; thus, enhancing their activity and causing serious side effects [7].

FCs are contained in citrus fruits together with polymethoxyflavones (PMFs) and coumarins (Cs), the latter also contained in several spices, especially cinnamon. Cs, Fs and PMFs share a similar chemical structure and are named with the common definition of oxygen heterocyclic compounds (OHCs).

All these chemical classes have been widely investigated for their wide range of biological activities. The health effects related to the consumption of PMFs are known enough to make them interesting nutraceuticals for dietary supplements [8].

Furocoumarins inhibit the calcium channel, reduce platelet aggregation [9], induce apoptosis in human promyelocytic leukaemia [10] and show hepatoprotective effects [11]. Imperatorin has been reported to have numerous potent pharmacological actions, including anti-inflammation [12], anti-bacterial [13], anti-allergic [14], beneficial cardiovascular effects [15,16,17], neuromodulation [18,19,20] and is able to induce apoptosis in human promyelocytic leukaemia and HL-60 cells [10]. Effects of angelicin against postmenopausal osteoporosis were demonstrated [21]. Bergapten and imperatorin also share in vitro anti-diabetic effects [22]. The FCs found in grapefruit juice show antioxidation, anti-inflammatory and inhibitory activities on breast cancer cell growth, especially bergaptol and bergapten [23]. Different components of bergamot oil showed in vitro activity against neuroblastoma cell growth [7].

Similarly, coumarins possess anti-inflammatory, anti-mutagenic, anti-tumorigenic and antioxidant properties [24,25,26], moreover have remarkable activities as antihistamine, spasmolysis, inhibition of insulin-induced lipogenesis, antibacterial [1] and anticancer, by acting as inhibitors of tumorigenesis as demonstrated in vivo [27,28] and in vitro [29]. However, in some cases, they showed hepatic [30,31] and pulmonary toxicity [32].

Indeed, as largely reported, Cs and FCs possess several beneficial properties. However, recent studies are focused on the evaluation of the adverse effects as a consequence of their dietary intake.

Among Cs, coumarin is the most investigated because of the harmful effects consequent to its dietary intake [32]. Currently, the legal provision concerning this field is the *Regulation (EC) No 1334/2008* of the European Parliament and of the Council of 16 December 2008 on flavourings and certain food ingredients with flavouring properties for use in and on foods, which includes only coumarin in Annex III, indicating the maximum level permitted in several foods (bakery ware, cereals and desserts) [33]. Most recently [34], the European Food Safety Authority (EFSA) concluded to maintain the tolerable daily intake (TDI) of 0.1 mg coumarin/kg [35] already established in the 2004 opinion [36]. According to the official regulation, the content of coumarin was already determined in many food matrices [31,37]. Whereas, for the Food and Drug Administration (FDA), “food containing any added coumarin as such or as a constituent of tonka beans or tonka extract is deemed to be adulterated” according to *Title 21—Food and Drugs Chapter I—Food and Drug Administration Department of Health and Human Services*.

No limits are imposed on the content of FCs in food, but as mentioned above, they can seriously affect drug metabolism due to the inhibition of the isoform CYP3A4 of cytochrome P450 [38] and the inhibition of efflux transporters [39]. The increase in bioavailability of many drugs can lead to cardiac disorders and respiratory depression [6]. Moreover, FCs have for a long time been known for their phototoxic reactions [40,41,42], such as phytophotodermatitis [43], due to the formation of intermediates which react with nucleophilic groups such as DNA, RNA and proteins [44].

The bioavailability of dietary FCs ranges from 54 to 81% [45] and there are many evidences about their distribution in the skin [46,47], meaning that also the oral administration of FCs may lead to skin pathologies after UV exposure.

Already in 1986, the International Agency for Research on Cancer (IARC) added 8-methoxypsoralen (8-MOP) into the group one list, as carcinogenic to humans. Despite the European Medicines Agency [48] and the European Food Safety Authority’s [49] suggestions, the European Parliament did not impose any limit of their content in food.

However, taking into account all the evidence cited above, the characterization of Cs and FCs in food and beverages is a topic of great interest. Until now, HPLC-DAD methods were widely applied for the quality control of citrus essential oils [50,51]. However, in derived food or cosmetics products, viz. for target analysis in complex samples, more sensitive and selective methods are needed, mainly based on the use of tandem MS systems that allow to achieve low limits of quantification (LOQ) [1,2,20,52,53,54,55].

The most comprehensive study about the content of FCs in food was carried out by Melough and co-workers, by using an UPLC-MS/MS system [54] to determine seven major FCs in 19 different foods, some of them already investigated [3].

The aim of the present work is to achieve the comprehensive determination of a total of 35 OHCs, among Cs, Fs and PMFs in different beverages, by using a novel HPLC-MS/MS method with an embebbed linear retention index (LRI) approach.

The LRI system is routinely used in gas chromatography since it has been introduced for the first time [56]; it was abandoned after a few attempts in LC due to a poor repeatability at inter-laboratory levels [57]. Recently, it was re-proposed in our laboratory for the identification of lipids [58] and OHC compounds [51,52,59] through LC methods combined with different detection systems, being confident about the highest batch-to-batch reproducibility of LC columns and instrumentations achieved in the last years. Indeed, the built LRI databases proved to be stable in different instrumental setups [51,52,55]. Within this context, the present research was aimed to characterize several citrus beverages and a marmalade sampled by HPLC-MS/MS; thus, combining the use of an MS/MS library and the LRI database for a reliable identification, even at trace level, below the limit of identification of the common UV detectors. Quantification was performed in Multiple Reaction Monitoring (MRM) mode by external calibration for all the 35 target OHCs. The obtained data add useful information to evaluate the FC dietary intake and the related risk assessment.

## 2. Materials and Methods

A total of 41 standards among OHCs and alkyl aryl ketones used as reference homologue series for the building of the LRI database were furnished by Merck Life Science (Merck KGaA, Darmstadt, Germany). The full names are provided in Table 1. Tetrahydrofuran (THF), ethyl acetate and ethanol, all HPLC grade, water and methanol both UHPLC-MS grade were provided by Merck Life Science. OHC solutions were prepared by using ethanol as a solvent, whereas alkyl aryl ketones were dissolved in acetonitrile. All standards and stock solutions were maintained at −18 °C before use.

### 2.1. Samples and Sample Preparation

Three bergamot alcoholic beverages, a lemon and a bergamot commercial juices, an Earl grey tea, a citrus infusion (composed of different dried fruits, not specified on the label) and a lemon marmalade were bought in a local market.

A home-made *limoncello* was prepared by ethanol maceration of the lemon peels for around 30 days, then the extract was added to water and sugar.

Juices were centrifuged at 3000 rpm for 5 min (bench top centrifuge, Neya 16R) to remove the pulp, then the supernatant was injected without further pre-treatment. All other samples were subjected to liquid–liquid extraction with ethyl acetate. In particular, 10 mL of alcoholic beverages and 150 mL of infusions were extracted with 10 mL and 50 mL of solvent, respectively. The extraction was carried out by manual shaking in a separatory funnel, the aqueous or hydro-alcoholic phase was iteratively extracted three times with the same amount of ethyl acetate. The three ethyl acetate phases were pooled and dried through a rotary evaporator (Envì EZ-2, Genevac, Ipswich, UK), applying a pre-set method called “low boiling point”.

Infusions were prepared by adding 150 mL of boiling water to one sachet, containing two grams of dried material. The infusion time was 5 min.

In the case of marmalade, 10 g of the product were weighed directly in a falcon, added to 10 mL of ethyl acetate and sonicated for 15 min (frequency, kHz 80); the mixture was filtered and the residue was extracted again. The procedure was repeated one more time.

The supernatans were pooled and dried through the Envì EZ-2 rotary evaporator, applying the “low boiling point” method.

All the residues were dissolved in 1 mL of ethanol before the HPLC-MS/MS analysis and three replicates were performed.

### 2.2. Instrumental and Analytical Conditions

The instrumental setup and the employed method were the same previously developed and validated [52].

The instrument was a Nexera X2 system coupled with a triple quadrupole mass spectrometer LCMS-8060 (Shimadzu, Duisburg, Germany) via an APCI interface set in positive ionization mode. The chromatographic system was equipped with two LC-30AD pumps, a SIL-30AC autosampler, a DGU-20A_5R_ degassing unit and a CTO-20AC oven. The separation was achieved by using an Ascentis Express C18 column (50 × 4.6 mm, 2.7 μm) provided by Merck. The mobile phase was A) water/methanol/THF (85:10:5, *v:v:v*) and B) methanol/THF (95:5, *v:v*). The chromatographic run was carried out at a flow rate of 2 mL min^−1^ and a temperature of 40 °C in gradient mode according to the following program: 0–4.5 min, 15–28% B; 4.5–7.0 min, 28–60% B; 7.0–11.0 min, 60–85% B, hold for 3 min. The injection volume was 2 μL.

The MS system operated in both full scan and MRM acquisition mode to ensure both untargeted and targeted analyses, in full scan and MRM mode, respectively. MS parameters were as follows: interface temperature was set at 450 °C; desolvation line (DL) and heat block temperatures were both 300 °C; nebulizing and drying gas flow were 3 and 15 L/min, respectively; the pressure of the CID gas was 270 kPa. The mass spectral range was 150–450 *m*/*z* for the untargeted analysis, while the targeted analysis was carried out in MRM mode through a synchronized method, which set specific acquisition windows according to the retention time of each target, and by applying a dwell time of 20.0 ms. In this way it was possible to obtain 10 scan per peak, as required for a correct quantification. Instead, the homologous series was analysed in Single Ion Monitoring positive mode (SIM+). The *m*/*z* monitored for the alkyl aryl ketones was: *m*/*z* 121, *m*/*z* 135, *m*/*z* 149, *m*/*z* 163, *m*/*z* 177, *m*/*z* 191 for acetophenone, propiophenone, butyrophenone, valerophenone, hexanophenone and heptanophenone, respectively. Target analytes (Cs, FCs and PMFs) were detected in MRM acquisition mode.

Taking into account the sensitivity of the MRM detection, the carry-over phenomenon was considered by injecting a reagent blank, represented by 2 μL of pure ethanol between two consecutive sample analyses, in order to evaluate the efficacy of the washing gradient step.

### 2.3. Qualitative and Quantitative Analysis

Peak identification was carried out by complementarily using the MS/MS library and the LRI database, as reported in the previous work [52]. The homologous series of alkyl aril ketones was injected before the samples and used for the calculation of the LRI values by means of the following equation, according to the Van den Dool and Kratz theory and as previously reported [51,52,59]:

(1) $$LRI=100\times \left[z+\frac{t_{Ri\;}-t_{Rz}}{t_{R\left(z+1\right)}-t_{Rz}}\right]$$

where *z* is the carbon number of the alkyl aryl ketone eluted before the analyte, *t_Ri_*, *t_Rz_* and *t_R_*_(*z*+1)_ are the retention times of the analyte and the alkyl aryl ketones eluted before and after the analyte.

A new software, namely ChromLinear (Chromaleont, Messina, Italy) version 1.0, developed ad hoc for LRI database handling in LC [60], was employed for a fast and automatic peak identification. The software is able to perform a dual-filter search, thus, excluding from the list of compounds with a high spectral similarity (minimum direct and reverse match were both set at a value of 800; they are calculated based on the NIST MS search algorithm), those falling out the window of ±4 LRI units.

The quantification was based on the creation of calibration curves built in MRM mode for all standards [52].

## 3. Results

**Figure 1:**
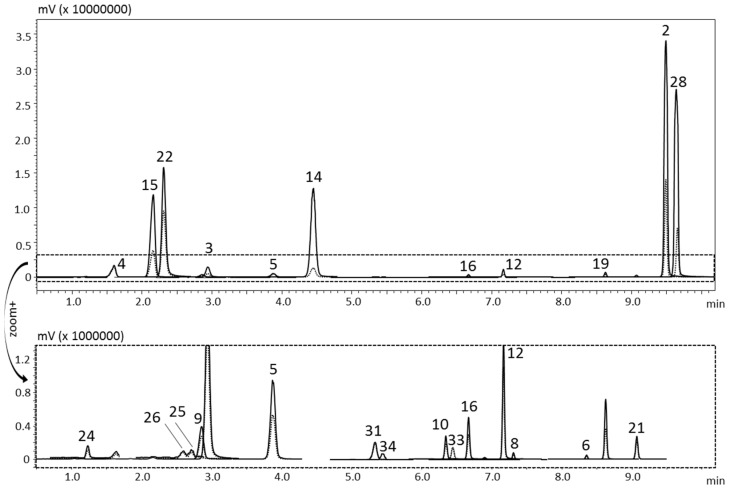
HPLC-MS/MS (MRM acquisition mode) chromatogram of the home-made *limoncello*. Quantifier ion, continuous line; qualifier ion, dotted line. For peak identification see Table 2.

**Figure 2:**
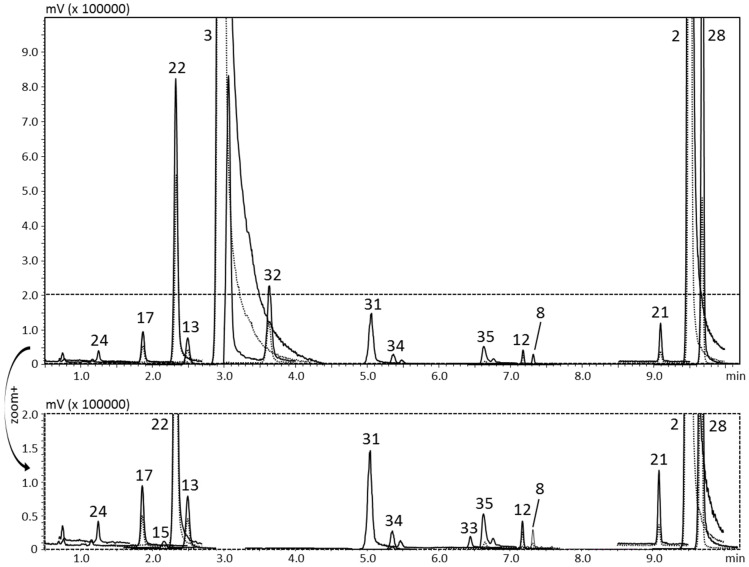
HPLC-MS/MS (MRM acquisition mode) chromatogram of the bergamot juice. Quantifier ion, continuous line; qualifier ion, dotted line. For peak identification see Table 2.

Figure 1 and Figure 2 show the qualitative profiles of two beverage samples, the home-made *limoncello* and the bergamot juice, respectively. The enlargement of the figure shows the components contained in a low amount, which were quantitatively determined through the sensitivity of this technique.

**Figure 3:**
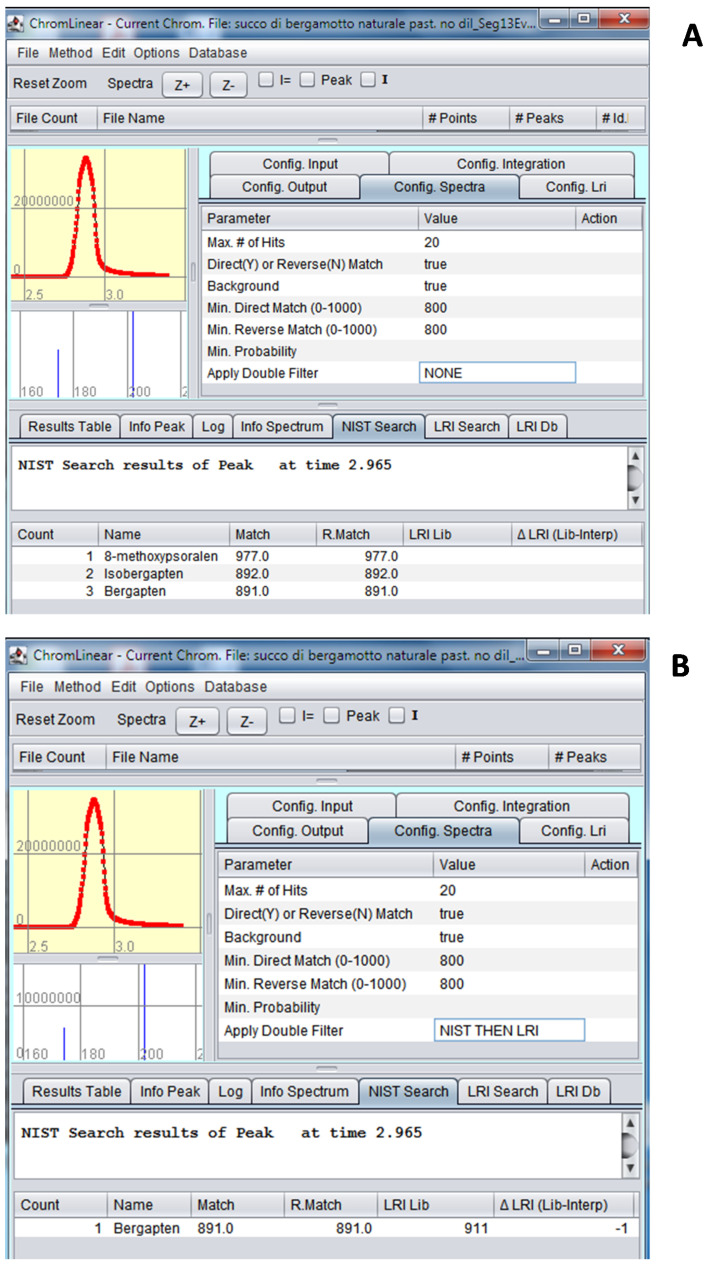
(**A**) Spectral similarity search results and (**B**) dual-filter search results for peak 3 (bergapten).

The full scan acquisition mode was used to verify potential co-elutions with the target compounds during the validation process. Table 1 reports the MRM transitions applied for the MS acquisition and the inter-sample LRI average for all the analytes in comparison with the tabulated values. The LRI system was applied for the unambiguous identification of target compounds as a library filter. A maximum difference of four units was obtained for all the compounds, thus, pointing out a satisfactory reproducibility of the LRI approach. For the first time, a novel software, *ChromLinear*, was applied to MS/MS analyses and the identification was automatically performed by match with MS/MS libraries in combination with the LRI database. As an example, Figure 3A shows the spectral similarity search results obtained for peak three (bergapten), pointing out that a list of three candidates was obtained, due to the fact that three isomers are characterized by quite identical fragmentation (same precursor and product ions, both quantifier and qualifier). The application of the additional LRI filter allows the univocal identification due to the automatic exclusion of two isomers characterized by a totally different retention index (Figure 3B). It is noteworthy that the use of the simple retention time could not represent a solution since at inter-laboratory levels or in different periods the retention time could be quite different, while it was already demonstrated that the LRI remains constant because of the normalization effect of the reference homologue series.

The validated method ensured a wide linearity range thanks to the application of the weighing factor, in order to correct the data heteroscedasticity. LOQ were in the range of 0.0003 and 0.009 mg L^−1^ for most compounds, except for a few exceptions (e.g., nobiletin, 0.0740 mg L^−1^). Recovery was satisfactory at four levels of concentration tested. More details about the validation parameters are available in the previous study by Arigò and co-workers [52].

Table 2 reports the amounts of OHCs contained in all the samples analysed.

In agreement with the study carried out by Dugo and co-workers [61], bergamot juice resulted in the richest sample with a total amount of 31 mg/L of OHCs, mostly represented by bergamottin and bergapten, 19 and 10 mg/L, respectively. Whereas the lemon juice showed a total of 1.64 mg/L among FCs, Cs and PMFs.

As for alcoholic drinks, the maceration of lemon flavedo in ethanol, as the traditional procedure to prepare *limoncello* in southern Italy, seems to extract a notable amount of OHCs. In particular, the most representative are FCs with a total of 24 mg/L, mainly 8-geranyloxypsoralen, bergamottin, biakangelicin, and oxypeucedanin hydrate (6.3, 3.9, 3.9 and 3.7 mg/L, respectively). Citropten (4.0 mg/L) and 5-geranyloxy-7-methoxycoumarin (2.9 mg/L) constitute almost the overall total for Cs, equal to 6.9 mg/L. The amount of biakangelicin, oxypeucedanin hydrate and citropten quantified in this sample of *limoncello* are very similar to those reported by Dugo [61] for another home-made *limoncello* samples.

Three different commercial bergamot alcoholic beverages were analysed in this study. The samples were purchased in local markets sited in Calabria, the only region where *Citrus bergamia* Risso plants are grown. Commercial *bergamino* samples gave very different results, pointing out the differences due to the production process or to the use of different ingredients. The samples showed a very different OHC composition with a total of 16.3, 7.5 and 3.5 mg/L for bergamot beverage A, B and C, respectively. In all cases, much of the total was represented by FCs, 14.0, 4.0 and 2.0 mg/L for sample A, B and C, respectively. Bergamottin and bergapten were the most abundant FCs in all products. Citropten was the major coumarin in all the samples (0.94, 0.58, 0.51 mg/L for sample A, B and C respectively), followed by 5-geranyloxy-7-methoxycoumarin (0.19, 0.16, 0.28 mg/L for sample A, B and C, respectively). Sample B is characterized by the presence of meranzin hydrate and isomeranzin in quite high amounts, 0.59 mg/L and 0.42 mg/L, respectively; both compounds were not detected in the other bergamot liqueurs.

PMFs were different both in qualitative and quantitative composition and the total amount was very low for all samples. Nobiletin was the most abundant PMF in sample B (0.92 mg/L) whereas sinensetin was the most present (0.97 mg/L) in sample A, followed by tetra-O-methylscutellarein (0.11 mg/L). Likely, OHCs extracted from the flavedo of citrus through a maceration process should reflect the OHC profiles of the corresponding peels than of the essential oils. In this regard, sample A was that one which better corresponds to the OHC composition of the bergamot cold-pressed essential oil [50,52].

A total of OHCs equal to 0.02 mg/L was quantified in the tea, whereas around 0.6 mg/L was found in the citrus peel infusion. Among the classes of OHC in the infusion, the main was PMFs.

**Figure 4:**
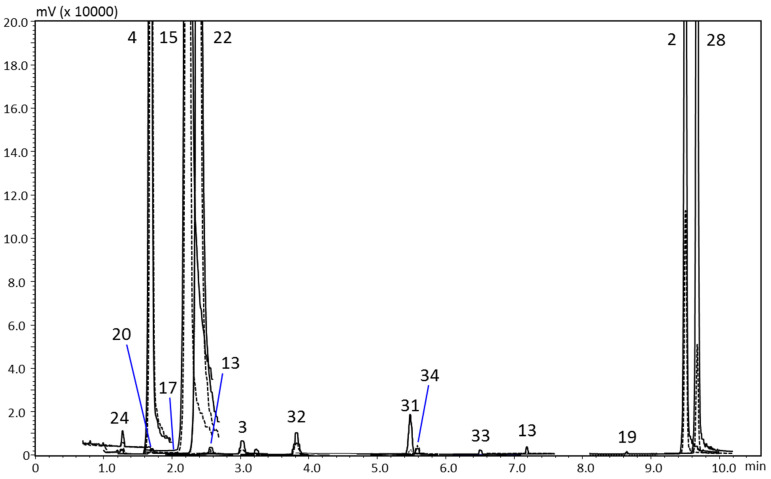
HPLC-MS/MS (MRM acquisition mode) chromatogram of the lemon marmalade. Quantifier ion, continuous line; qualifier ion, dotted line. For peak identification see Table 2.

Table 2 also reports the quantitative data for the lemon marmalade, while Figure 4 shows its MRM chromatographic profile. Byakangelicin is the most abundant compound (16.3 mg/kg) and together with oxypeucedanin hydrate (12.4 mg/kg) represents the main constituents of the FC class, resulting in a total of 29.9 mg/kg. Citropten is the main coumarin (13.5 mg/kg) and some PMFs are contained, but in a low amount.

Recently, the same approach was successfully applied to characterize OHCs in flavoured citrus beer, also in order to prevent food fraud by comparing the volatile profile of the samples with the not volatile composition [52].

## 4. Discussion

Several scientific articles conclude that there is a lack concerning the in-depth characterization of FCs in foodstuff [40,41,54,62,63].

The object of this research was based on the analysis of several drinks, which can cause the ingestion of considerable amounts of FCs.

The analytical strategy applied in this study was the HPLC-MS/MS method previously developed and successfully applied for the quality control of FCs in beers and cosmetics [52,55]. The sensitivity of the MRM acquisition mode guarantees the correct quantification of OHCs at a very low concentration level. Moreover, the main novelty of this approach arises in the use of the LRI system in combination with MS libraries for the rapid and unambiguous identification of the targets.

We focused the sampling mainly on citrus drinks because OHCs are contained especially in the fruit of this botanical genus. The OHC profile is different depending on the species and the part of the fruit considered, i.e., peel or pulp.

The bergamot fruit is used mainly for the production of essential oil, then the rest of the fruit is generally considered waste [64]. However, in the last years, the production of bergamot beverages is increasing and the consequent dietary intake of FCs should be carefully evaluated.

In order to characterize bergamot fruit and its by-products, in 2016 Russo and co-workers [64] reported a total amount of OHCs in bergamot juice equal to 60 mg/kg, where bergapten and bergamottin were 22 and 37 mg/kg, respectively. The total amount of OHCs in peels was equal to 474 mg/kg, mainly represented by bergapten and bergamottin 219 and 217 mg/kg, respectively.

Flavoured bergamot drinks can be produced by using the essential oil, the juice or the peel of the fruit as ingredients of the preparation, leading to a different content of OHCs. For instance, the maceration of bergamot peels with ethanol, as a traditional method to prepare *bergamino* liqueur, leads to a high content of FCs, as a consequence of the bergapten and bergamottin amounts in the flavedo. Often, commercial alcoholic beverages are not produced by maceration of the bergamot peels, but through the addition of distilled or cold-pressed bergamot essential oils, in this case the amount of OHCs is directly related to the type and the amount of the essential oil used. When distilled essential oils are used, the FC content should be negligible, while for cold-pressed essential oil the content of FCs could grow up to thousands of mg/L.

The same consideration can be applied to other types of beverages flavoured with different species of citrus essential oils (lemon, lime, grapefruit), with the exception of beverages flavoured with orange and mandarin essential oils [8,65] that do not contain FCs.

According to the last toxicological evaluation [38,39,40,41,42], the quality control of these compounds in food represents an emerging subject and the development of an analytical method suitable for this purpose is a challenge. The information derived by the characterization of several types of beverages provides a starting point to discuss the dietary intake of FCs and below which value it can be considered safe.

Versari and co-workers performed the characterization of twelve *Limoncello* liqueurs provided from commercial sources; only six OHCs were detected and quantified [66]. The most recent evaluation of the FC amount contained in a variety of citrus drinks was realized by Gorgus and co-workers [67]. The dietary intake of FCs caused by the ingestion of grapefruit juice was estimated to be higher compared to those caused by the ingestion of other citrus-containing beverages. However, only a few compounds were investigated in this study [67].

Our group already carried out research about the determination of OHCs in citrus products by HPLC with UV detection [61]. In that study, different citrus beverages were analysed, but the results obtained were strictly related to the low sensitivity of the detector employed. In particular, 27 OHCs were investigated in a commercial Earl Grey tea, two laboratory-made juices (lemon and bergamot), a home-made *limoncello* and two commercial *bergamino* liquors. *Limoncello* resulted the qualitatively richest, whereas the bergamot juice was surprisingly the most abundant from the quantitative point of view. This depends on the ingredients used for the commercial drinks; in fact, despite the traditional preparation being based on the maceration of flavedo with alcohol which should correspond to the high extraction of FCs, the industrial products may be prepared by using essential oils or juices, explaining the lower amounts of FCs detected in *limoncello* compared to the bergamot juice.

The variables responsible for the differences in the OHC quali-quantitative profile of the analysed samples are numerous. The ingredients of the preparation play the key role on the target composition in the samples. In facts, whereas home-made *limoncello* and *bergamino* are prepared with the traditional recipe which is based on the flavedo maceration with ethanol, the commercial liqueurs may contain essential oils (cold-pressed or distilled), alcoholic extracts and/or juice.

As for infusion products, the quantification of OHCs, especially FCs, has attracted special interest, following a case report published in *The Lancet* journal in 2002 [68]. In this article, the abundant and continuous assumption of earl grey tea was clearly connected with neurologic adverse effects. The reason was attributed to bergapten, which is contained in the bergamot essential oil added to black tea, to confer the characteristic aroma. The author’s conclusion was that “even tea may lead to health problems if flavoured and consumed in extraordinarily high quantities”.

In this research, an earl grey tea and a citrus fruit infusion were also taken into account. Both samples were extracted prior to the analysis in order to concentrate the target compounds, which are contained in low amounts mainly because of their low water solubility, then the calculated concentrations (above the LOQ) were corrected for the dilution factor. The infusion of dried citrus peels resulted richer, than the earl grey tea, both in FCs and total OHCs. This means that the powder could be composed mainly of dried peels of mandarin and orange fruits. The reason is that often, to avoid the presence of FCs, peels of sweet orange or mandarin fruits are preferred, also for the lower price and especially for their sweet taste compared to other types of citrus fruit.

In this sample of earl grey tea, the amounts of bergamottin and bergapten were very low. The ingredients of the product, in particular the essence used to flavour the black tea, were not specified on the label, as it often happens, but we can suppose that the producers used cold-pressed essential oils, which justifies the presence of OHCs.

To summarize, among the beverages analysed, bergamot juice was the richest in FCs, followed by the home-made *limoncello*, with 29 and 24 mg/L, respectively.

Moreover, for the absolute amounts of the targets in the samples, it is important to consider the type of drink, which is correlated with the volume generally ingested.

For instance, the consumption of 20 mL of *limoncello* makes 0.6 mg of FCs, but drinking 200 mL of juice causes the makes 4.8 mg of FCs.

A dietary intake of around 20 g of marmalade causes the ingestion of 0.6 mg of FCs.

## 5. Conclusions

Due to the lack of detailed data about the content of FCs and Cs in food, the opinions regarding the maximum admitted level are still varied and contrasting, consequently an official regulation is still missing.

This HPLC-MS/MS method, characterized by the application of the LRI system combined with the MS libraries, was successfully applied to determine three different classes of OHCs in different citrus beverages and marmalade, representing a significant step forward with respect to previous works which are mainly focused on a limited number of compounds. The highest sensitivity of the MRM method allowed for the detection of a major number of compounds, thus, resulting in a more accurate evaluation of the total amount of FCs and Cs. Their quantification represents the main purpose of the present research, aimed to add data that can be used to estimate their dietary intake.

The method will be applied to other food matrices and new analytical technologies, as supercritical fluid chromatography coupled to triple quadrupole mass spectrometry, will be applied and compared with the present method.

## Figures and Tables

**Table 1:** MRM conditions (transition, voltages and collision energies). Q, quantifier ion; q, qualifier ion; CE, collision energy, and inter-sample LRI average (Δ LRI) for all the analytes in comparison with tabulated values. SIM, Single Ion Monitoring.

Compound	Class	MRM Transitions			Tabulated LRI	Inter-Sample LRI Average	Δ LRI
		(M + H)^+^	Q (CE)	q (CE)			
**Target compounds**							
Meranzin hydrate	C	279	189 (−17)	261 (−7)	783	781	2
Herniarin	C	177	121 (−21)	77 (−25)	799	801	2
Byakangelicin	FC	317 334.7	231 (−19)	233 (−13) 174.95 (−31)	825	824	1
8-methoxypsoralen	FC	217	202 (−21)	174 (−25)	837	830	3
Psoralen	FC	187	131 (−21)	77 (−40)	842	844	2
Angelicin	FC	187	131 (−25)	77 (−35)	853	854	1
Oxypeucedanin hydrate	FC	305	203 (−20)	147 (−32)	864	861	3
Citropten	C	207	192 (−20)	163 (−15)	874	874	0
Isopimpinellin	FC	247	217 (−25)	232 (−18)	884	886	2
Meranzin	C	261	189 (−15)	131 (−29)	886	885	1
Isomeranzin	C	261	189 (−17)	131 (−30)	900	900	0
Heraclenin	FC	287	203 (−17)	147 (−33)	904	907	3
Bergapten	FC	217	202 (−19)	174 (−25)	911	912	1
Sinensetin	PMF	373	343 (−30)	312 (−21)	937	938	1
Isobergapten	FC	217	202 (−21)	174 (−26)	940	942	2
Byakangelicol	FC	317	218 (−29)	175 (−25)	950	954	4
Oxypeucedanin	C	287	203 (−18)	59 (−38)	974	971	3
Nobiletin	PMF	403	373 (−34)	327 (−31)	1016	1017	1
Tetra-O-methylscutellarein	PMF	343	313 (−30)	282 (−25)	1027	1029	2
Trioxsalen	FC	229	142 (−25)	173 (−22)	1076	1075	1
Imperatorin	FC	271	203 (−15)	147 (−31)	1082	1079	3
Tangeretin	PMF	373	343 (−30)	211 (−34)	1087	1085	2
Epoxyaurapten	C	315	163 (−16)	107 (−25)	1096	1096	0
5-O-demethylnobiletin	PMF	389	359 (−30)	341 (−27)	1100	1102	2
Phellopterin	FC	301	233 (−14)	218 (−30)	1104	1105	1
Cnidilin	FC	301	233 (−15)	218 (−28)	1129	1126	3
Gardenin A	PMF	419	389 (−32)	371 (−28)	1130	1134	4
Isoimperatorin	FC	271	203 (−15)	147 (−31)	1159	1160	1
Epoxybergamottin	FC	355	203 (−18)	215 (−19)	1166	1162	4
Gardenin B	PMF	359	329 (−29)	311 (−25)	1172	1170	2
Cnidicin	FC	355	219 (−16)	173(−32)	1303	1305	2
8-geranyloxypsoralen	FC	339	203 (−25)	95 (−25)	1316	1316	0
Aurapten	C	299	163 (−15)	107 (−40)	1336	1339	3
Bergamottin	FC	339	203 (−14)	147 (−35)	1355	1358	3
5-geranyloxy-7-methoxycoumarin	C	329	193 (−20)	149 (−25)	1364	1366	2
**Homologous series**		**SIM** **(M − H)^+^**					
Acetophenone	AAK	121			800		
Propiophenone	AAK	135			900		
Butyrophenone	AAK	149			1000		
Valerophenone	AAK	163			1100		
Hexanophenone	AAK	177			1200		
Heptanophenone	AAK	191			1300		

**Table 2:** Amount of OHCs (mg/L), with the corresponding standard deviation, in the samples analysed. Standard deviation less than 0.001 was not reported. LOQ, limit of quantification.

Compound	Class	Alcoholic Beverages				Infusions		Commercial Juices		Lemon Marmalade
		Home-Made *Limoncello*	Bergamot Beverage A	Bergamot Beverage B	Bergamot Beverage C	Earl grey	Citrus Fruits	Bergamot	Lemon	
Angelicin (1)	FC									
Bergamottin (2)	FC	3.93 ± 0.67	3.50 ± 0.25	2.04 ± 0.02	1.28 ± 0.07	0.001	0.001	19.41 ± 0.91	0.32 ± 0.002	1.16 ± 0.065
Bergapten (3)	FC	0.11 ± 0.007	9.18 ± 0.64	2.03 ± 0.02	0.31 ± 0.01	0.01	0.008	9.74 ± 0.26	<LOQ	<LOQ
Byakangelicin (4)	FC	3.91 ± 0.3		0.11 ± 0.001	0.12 ± 0.004	0.0004	0.01		0.36 ± 0.02	16.3 ± 2.35
Byakangelicol (5)	FC	1.29 ± 0.3			0.06 ± 0.002		0.0003			
Cnidicin (6)	FC	0.14 ± 0.01			0.01 ± 0.001		<LOQ		0.04	
Cnidilin (7)	FC	0.01 ± 0.002			<LOQ	<LOQ	<LOQ		<LOQ	
Epoxybergamottin (8)	FC	0.40 ± 0.003	1.26 ± 0.003		0.003	<LOQ	0.0001	0.03 ± 0.001		
Heraclenin (9)	FC	0.79 ± 0.14			0.01	0.0003				
Imperatorin (10)	FC	0.01 ± 0.002			0.002				0.01 ± 0.004	
Isobergapten (11)	FC									
Isoimperatorin (12)	FC	0.16 ± 0.007			0.01	<LOQ	0.00005	0.01 ± 0.001	0.01	<LOQ
Isopimpinellin (13)	FC	0.003	0.001	0.002	0.002	0.0001	0.00002	0.01	<LOQ	<LOQ
Oxypeucedanin (14)	FC	2.25 ± 1.42		<LOQ	0.11 ± 0.004					
Oxypeucedanin hydrate (15)	FC	3.69 ± 0.22	0.06 ± 0.001	0.07 ± 0	0.10 ± 0.004	0.0004	0.008	0.03	0.22 ± 0.003	12.4 ± 3.01
Phellopterin (16)	FC	0.80 ± 0.008		<LOQ	0.02 ± 0.001		<LOQ		<LOQ	
Psoralen (17)	FC		0.01 ± 0.001	<LOQ	<LOQ	0.0003	<LOQ	0.06 ± 0.001		<LOQ
Trioxsalen (18)	FC									
8-geranyloxypsoralen (19)	FC	6.34 ± 0.39		<LOQ	0.19 ± 0.002		0.0003		0.13 ± 0.01	<LOQ
8-methoxypsoralen (20)	FC			<LOQ	<LOQ	<LOQ				<LOQ
**Tot of FC**		23.9 ± 3.47	14.1 ± 0.89	4.26 ± 0.04	2.22 ± 0.1	0.01	0.03	29.3 ± 1.17	1.08 ± 0.04	29.86 ± 5.43
Aurapten (21)	C	0.02 ± 0.002	0.001	<LOQ	0.003 ± 0		<LOQ	0.03 ± 0.001		
Citropten (22)	C	3.98 ± 0.39	0.94 ± 0.11	0.58 ± 0.003	0.51 ± 0.02	0.008	0.01	0.78 ± 0.01	0.18 ± 0.01	13.5 ± 2.1
Epoxyaurapten (23)	C									
Herniarin (24)	C	0.01 ± 0.002	0.01 ± 0.001	0.03	0.02	0.003	0.0002	0.01	<LOQ	<LOQ
Isomeranzin (25)	C	<LOQ		0.42	<LOQ		0.03			
Meranzin (26)	C	<LOQ		<LOQ	<LOQ		0.0004			
Meranzin hydrate (27)	C			0.59 ± 0.03		0.001	0.03			
5-geranyloxy-7-methoxycoumarin (28)	C	2.88 ± 0.24	0.19 ± 0.02	0.16 ± 0.003	0.28 ± 0.007	0.0001	0.0004	0.54 ± 0.01	0.27 ± 0.02	0.54 ± 0.06
**Tot of C**		6.89 ± 0.63	1.14 ± 0.12	1.77 ± 0.03	0.81 ± 0.02	0.01	0.08	1.36 ± 0.02	0.46 ± 0.03	14.04 ± 2.16
Gardenin A (29)	PMF			<LOQ	0.02		0.003	0.07		
Gardenin B (30)	PMF			<LOQ	<LOQ	<LOQ	<LOQ			
Nobiletin (31)	PMF	<LOQ	0.05 ± 0.006	0.92 ± 0.01	0.3 ± 0.02	0.001	0.4 ± 0.01	0.07 ± 0.001	0.02	0.45 ± 0.024
Sinensetin (32)	PMF		0.97 ± 0.01	0.14 ± 0.002	0.02 ± 0.002	0.0002	0.05	0.33 ± 0.01		0.17 ± 0.023
Tangeretin (33)	PMF	0.063	0.07 ± 0.001	0.13 ± 0.001	0.04	0.0003	0.02	0.04 ± 0.001	0.03	0.31 ± 0.001
Tetra-O-methylscutellarein (34)	PMF	<LOQ	0.11 ± 0.001	0.11	0.04 ± 0.002	0.0004	0.02	0.06		0.55 ± 0.004
5-O-demethylnobiletin (35)	PMF			0.13	0.02	0.0004	0.01	0.09 ± 0.003	0.05	
**Tot of PMF**		0.06	1.20 ± 0.02	1.43 ± 0.01	0.44 ± 0.03	0.002	0.48 ± 0.01	0.65 ± 0.01	0.10	1.48 ± 0.052
**TOT**		30.8 ± 4.13	16.3 ± 1.04	7.46 ± 0.09	3.47 ± 0.15	0.02	0.59 ± 0.01	31.3 ± 1.20	1.64 ± 0.07	42.7 ± 7.64

## Data Availability

Not applicable.
